# Origin, enzymatic response and fate of dissolved organic matter during flood and non-flood conditions in a river-floodplain system of the Danube (Austria)

**DOI:** 10.1007/s00027-013-0318-3

**Published:** 2013-10-22

**Authors:** Anna Sieczko, Peter Peduzzi

**Affiliations:** Department of Limnology and Oceanography, University of Vienna, Althanstrasse 14, 1090 Vienna, Austria

**Keywords:** Dissolved organic matter, Bacteria, Extracellular enzymatic activity, Floodplain

## Abstract

Spectroscopic techniques and extracellular enzyme activity measurements were combined with assessments of bacterial secondary production (BSP) to elucidate flood-pulse-linked differences in carbon (C) sources and related microbial processes in a river-floodplain system near Vienna (Austria). Surface connection with the main channel significantly influenced the quantity and quality of dissolved organic matter (DOM) in floodplain backwaters. The highest values of dissolved organic carbon (DOC) and chromophoric DOM (CDOM) were observed during the peak of the flood, when DOC increased from 1.36 to 4.37 mg l^−1^ and CDOM from 2.94 to 14.32 m^−1^. The flood introduced DOC which consisted of more allochthonously-derived, aromatic compounds. Bacterial enzymatic activity, as a proxy to track the response to changes in DOM, indicated elevated utilization of imported allochthonous material. Based on the enzyme measurements, new parameters were calculated: metabolic effort and enzymatic indices (EEA 1 and EEA 2). During connection, bacterial glucosidase and protease activity were dominant, whereas during disconnected phases a switch to lignin degradation (phenol oxidase) occurred. The enzymatic activity analysis revealed that flooding mobilized reactive DOM, which then supported bacterial metabolism. No significant differences in overall BSP between the two phases were detected, indicating that heterogeneous sources of C sufficiently support BSP. The study demonstrates that floods are important for delivering DOM, which, despite its allochthonous origin, is reactive and can be effectively utilized by aquatic bacteria in this river-floodplain systems. The presence of active floodplains, characterized by hydrological connectivity with the main channel, creates the opportunity to process allochthonous DOC. This has potential consequences for carbon flux, enhancing C sequestration and mineralization processes in this river-floodplain system.

## Introduction

River-floodplain systems often have a broad range of lotic, lentic and semi-aquatic habitats with high species richness (Ward and Stanford 2006). Such environmental diversity creates opportunities for numerous linkages between the aquatic and terrestrial ecosystem and promotes interactions between abiotic factors and biotic processes. These couplings have received insufficient attention, particularly the linkage between the hydrological cycle and the carbon cycle (Battin et al. 2008). In particular, floodplain backwaters are seldom included in calculations of global carbon flux; they play a significant role in organic matter cycling and carbon mineralization, sequestration and transport (Battin et al. 2008).

One of the dominant forms of organic material in aquatic environments, including floodplains, is dissolved organic matter (DOM). DOM is a heterogeneous mixture of molecules, some of which originate from the surrounding area (allochthonous DOM), while others are generated within the system (autochthonous DOM). Allochthonous input of organic material is considered to be the dominant substrate supply for microorganisms in inland waters (Tranvik and Jansson 2002). Most DOM of terrestrial origin is believed to enter streams (Royer and David 2005), tropical rivers (Wiegner et al. 2009) and floodplains (Peduzzi et al. 2008) during seasonal floods and storms.

Bacterial utilization of DOM can be highly variable, depending on the lability of the organic matter. In rivers (del Giorgio and Pace 2008) and floodplains (Mladenov et al. 2007), bacterial metabolism may be supported by two different pools: quickly cycling DOM together with more slowly utilized semi-labile DOM. The highly labile pool, typically linked to phytoplankton development, is often rapidly exhausted. In contrast, the semi-labile pool appears to be strongly coupled to terrestrial DOC, which increases after flood events (del Giorgio and Pace 2008). This implies that aquatic food webs are coupled to watershed inputs of organic carbon (Pace et al. 2004). Some lake carbon cycles are reported to be heavily subsidized by organic carbon from the surrounding landscape (Carpenter et al. 2005; Cole et al. 2011). The importance of terrestrial material for bacterial metabolism has been emphasized in boreal headwater streams (Berggren et al. 2009), temperate lakes (Cole et al. 2006; Carpenter et al. 2005; Pace et al. 2004), oligotrophic humic lakes (Daniel et al. 2005) and estuaries (Van den Meersche et al. 2009).

Aquatic heterotrophic bacteria may act as links that transfer organic carbon to higher trophic levels, and/or sinks that mineralize DOM, resulting in CO_2_ production. Numerous studies have coupled bacterial respiration to allochthonous sources, underlining that little of the terrestrial C is passed to higher trophic levels (Cole et al. 2002; Kritzberg et al. 2005; Karlsson et al. 2007). Other evidence, however, suggests that autochthonous organic matter may be significantly respired as well (McCallister and Giorgio 2008). Among the many environmental factors (nutrient availability, temperature, photochemical impacts) (McCallister et al. 2005; Apple et al. 2006) that influence substrate partitioning between biomass generation and respiration, also seasonal disturbance events (floods, storms etc.) clearly affect bacterial processes. Flood pulses deliver substantial amounts of bioavailable DOM that may stimulate bacterial metabolism, for example in tropical rivers (Wiegner et al. 2009), streams (Ågren et al. 2008) and lakes (Lennon and Cottingham 2008). These sudden inputs of different carbon sources may increase microbial productivity (Docherty et al. 2006), respiration and rates of biogeochemical cycles (Kritzberg et al. 2005). Hydrological connectivity appears to be a crucial driving force in river-floodplain systems as well, influencing the quantity and quality of DOM and the related microbial processes (Schiemer et al. 2006; Mladenov et al. 2007; Peduzzi et al. 2008). In such highly variable water bodies, DOM bioavailability and the fate of utilized DOM, i.e. its partitioning into bacterial catabolic and anabolic processes, remains largely unresolved. Therefore, the factors determining DOM bioavailability in river-floodplain systems need to be better understood, as does the balance between anabolic and catabolic processes.

Losses and changes of organic matter due to microbial degradation have been assessed in several ways. One approach applies spectroscopic techniques to characterize and trace DOM dynamics (McKnight et al. 2001; Weishaar et al. 2003). This method relates the optical properties of DOM to its chemical composition, thus inferring DOM bioavailability. Nonetheless, characterizing DOM based solely on absorbance and fluorescence techniques has drawbacks. These include overlooking the highly reactive and quickly cycling DOM pool rapidly metabolized by microbial activity and also the non-chromophoric DOM. When faced with diverse DOM sources, bacteria produce a broad array of enzymes (Chróst 1991). Therefore, techniques that measure extracellular enzymatic activity (EEA) link bacterial activity to DOM composition and allow carbon sources and utilization patterns to be examined more closely (Foreman et al. 1998; Romaní et al. 2006). This approach may be especially valuable to study the bacterial response to rapidly released DOM during floods (Burns and Ryder 2008). We combined spectroscopic and enzymatic approaches, together with bacterial secondary production (BSP), to elucidate flood-pulse-linked differences in C sources and the related microbial processes. First, we asked whether surface connection with the main Danube channel significantly influenced DOM quantity and quality in floodplain backwaters. We then explored whether flooding mobilized a significant fraction of the semi-labile DOM from the catchment, which could then promote bacterial metabolism. We also attempted to understand whether metabolized DOM resulted more in catabolic or more in anabolic processes in relation to DOM origin and hydrological changes in river-floodplain systems. The focus was on the importance and availability of allochthonous DOM for microbial utilization.

## Methods

**Fig. 1:**
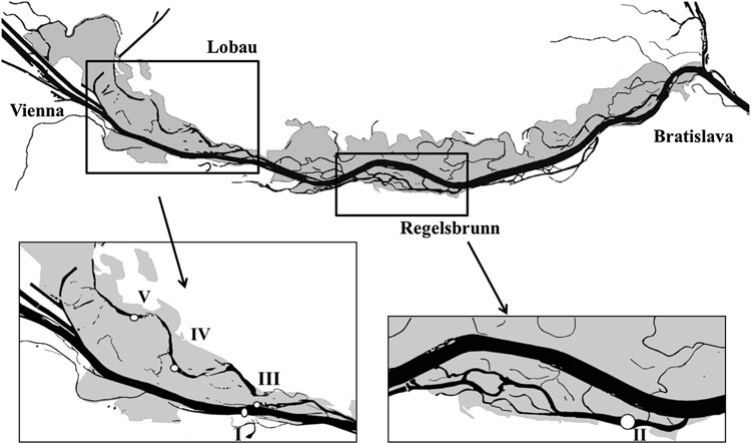
Study site: main channel of the Danube and its floodplains downstream of Vienna (side-arm system of Lobau and Regelsbrunn). *Circles* and *numbers* indicate sampling stations

The Danube is one of the longest rivers in Europe. The river and its floodplain characteristics are described in more detail in Heiler et al. (1995) and Schiemer et al. (1999a). Our investigation was conducted in the main channel of the Danube (I) and two sections of the “Danube Floodplain National Park” near Vienna, Austria (Fig. 1). Although the entire river segment is strongly impacted by regulation, it represents one of the very few remaining semi-natural river-floodplain systems in central Europe. It offers a broad spectrum of river-floodplain connections from strongly dammed sites to areas exhibiting a high degree of hydrological connectivity. Within this floodplain area, four stations were selected. They display diverse hydrological characteristics representing a gradient of connectivity to the main channel (Table 1). Two dynamically connected side arms were chosen: one (II) located in section more isolated from the main channel (called Lobau), another (III) situated in recently reconnected section (Schiemer et al. 1999b). At a high water level in the river, both locations experience direct through-flow, changing the conditions from disconnected to connected. Stations designated as IV and V (Fig. 1) are located farther from the main channel (Table 1) in a semi-separated section of the floodplain; they are severed from direct through-flow by a levee and additional weirs. This results in connection to the Danube only at high flood (approx. 18 days per year, Federal Waterway Agency). During previous years, an average flood lasted for ~10 days and usually occurred in May–June (Tockner et al. 1999; Hein et al. 2003; Peduzzi et al. 2008); the years when no flood occurred were also recorded (Preiner et al. 2008).

**Table 1:** Yearly average surface hydrological connectivity and distance from main stem for the study locations (II, III, IV, V)

Station	Location	Distance from the connection point with the main stem of the Danube (m)	Average yearly surface connectivity to the Danube (days)
II	Mannsdorfer Hagel	50	138
III	Regelsbrunn	300	220
IV	Kühwörther Wasser	820	18
V	Mittelwasser	1,680	18

Since the investigated backwaters are relatively shallow pools (0.63–1.5 m) and usually well mixed by wind and currents (Hein et al. 1999), each water body was sampled at a selected point known to be representative for the respective area. Surface water samples were collected on 16 dates from May–October 2009, typically monthly but intensified during changing hydrological situations (e.g. flood events, receding water level) to 2–3-day intervals. Water level and temperature were measured in situ at each location. The water samples were taken with rinsed polyethylene bottles (10 l) and transported to the laboratory within 3 h in dark boxes at in situ temperature. Three nitrogen compounds—nitrate, ammonia and dissolved organic nitrogen (DON) and soluble reactive phosphorus (SRP)—were determined within 24 h of collection. The water samples were analyzed following German standard methods for the examination of water: nitrate: DIN 38405-29, ammonia: DIN 38406-5, SRP: DIN EN ISO 6678. DON was calculated by subtracting ammonia from soluble Kjeldahl-N (Peduzzi et al. 2008). The method DIN 38406-5 was applied to obtain soluble Kjeldahl-N.

### Hydrology

The hydrological status was described by applying an “extent of connection” parameter (EC), defined as the deviation in meters from the level of connection [m] between the main channel and the respective floodplain section (Peduzzi et al. 2008). The degree of connectivity was expressed as the difference in height between the water level of the river at the time of sampling and a given side arm-specific topographic level at which inflow conditions are established. The amount of riverine water input into the floodplain section or the degree of isolation is a function of EC, which is related to the Danube water level. This value can be either positive (surface connection established to some degree), zero or negative (no surface connection). The EC-values of four locations (II, III, IV, V) were tested for significant differences in water level between connected and disconnected phases (*p* < 0.001). Hence, the measured parameters were split into 3 data sets: (1) data collected from stations II, III, IV and V during surface connection with the main river and (2) data obtained from stations II, III, IV and V when no surface connection existed (henceforth termed connected and disconnected, respectively). The third data set contains only Danube data obtained from the whole sampling period.

### Dissolved organic matter properties

Dissolved organic matter quantity and quality was analyzed by filtering water through pre-combusted (500 °C, GF/F, 0.7 μm, pore size) filters within 3 h of collection. DOC concentration [mg l^−1^] was obtained by high-temperature combustion using a Shimadzu TOC 5000 analyzer (Benner and Strom 1993), and the ratio DOC/DON was determined. The optical properties of DOM were investigated to obtain proxies for DOM quantity and quality. The absorption coefficient at 350 nm (a_350_) was chosen to describe changes in CDOM quantity (Kowalczuk et al. 2003; Para et al. 2010). The a_350_ [m^−1^] was obtained using the relationship: a350 = 2.303 A(350 nm)/L, where L is path length (0.01 m). To obtain an aromaticity index of DOC, carbon specific UV absorbance (SUVA_254_) was calculated. SUVA_254_ [l mg^−1^ m^−1^] is the ratio of absorption at 254 nm to the DOC concentration and is positively correlated with DOC aromaticity (Weishaar et al. 2003). To classify the origin of DOM, the fluorescence index (FI), proposed by McKnight et al. (2001), was determined with a Shimadzu spectrofluorophotometer RF-5301 PC. FI is the ratio of the emission intensity at 450 to 500 nm under excitation at 370 nm. The index allows distinguishing DOM originating from microbial sources (including algal derived) from terrestrially derived DOM. An endpoint index value of ~1.3 indicates allochthonously produced DOM, whereas an index of ~2.0 implies autochthonously derived DOM. This method was already successfully applied in Danube waters (Peduzzi et al. 2008).

### Bacterial parameters

All bacterial measurements were performed on unfiltered samples. Bacterial abundance (BA) was determined from the formaldehyde-fixed water sample (1–2 ml), which was stained with 4,6-diamidino-2-phenylindole-dihydrochloride (DAPI) (10 μg ml^−1^); bacterial cells were enumerated by epifluorescence microscopy (Nikon E 800) in 20–30 randomly chosen fields according to (Porter and Feig 1980) with a minimum of 500–600 cells counted (Noble and Fuhrman 1998). Bacterial secondary production (BSP) was measured based on the incorporation of [^3^H] thymidine (20 μl, 0.1 mCi ml^−1^ final concentration) in three replicate samples; two formaldehyde-killed samples served as controls (Fuhrman and Azam 1982). BSP [μgC l^−1^ h^−1^] was assessed using a Danube-specific conversion factor of 3.2 × 10^18^ cells produced per mol of thymidine incorporated (Berger et al. 1995).

The activity of three hydrolases: α-, β- glucosidases and leu-aminopeptidase (EEAa, EEAb, EEAleu, respectively) was calculated using fluorogenic substrates: 4-methylumbelliferyl (MUF)-α-D-glucoside, 4-MUF-β-D-glucoside and 4-methyl-7-coumarinylamide (MCA)-leucine; final concentration 0.5 mmol. Fluorescence (excitation at 360 nm, emission at 444 nm) was measured with a spectrofluorophotometer (Shimadzu RF-5301 PC) at (t_0_) and after incubation in the dark (t_1_) at in situ temperature and results were calculated as nmol substrate hydrolyzed per hour (Hoppe 1993). Phenol oxidase (PhOx) activity was determined using L-3.4-dihydroxyphenylalanine (L-DOPA) as a substrate (Pind et al. 1994). Two ml of unfiltered sample water and MilliQ water (control) were mixed with 2 ml of DOPA stock solution (5 mM DOPA in 2.5 mM NaHCO_3_ buffer, pH 8.3). The absorbance was measured at 460 nm in a spectrophotometer (Hitachi U-2000) immediately after adding the substrate (t_0_) and after incubation in dark and at in situ temperature for 180–220 min (t_1_). Activity of PhOx [nmol product h^−1^] was calculated using Beer’s Law and the molar absorbancy coefficient for the L-DOPA product 3-dihydroindole-5.6-quinone-2-carboxylate (diqc) (3.7 × 10^4^) (Mason 1948).

### Statistical data analyses

The data were processed using Microsoft Excel. Data were tested for normal distribution and homogeneity of variances with R 2.11.1 and SPSS 17.0 for Windows. Parametric tests were applied because data were, for the most part, normally distributed (Independent samples *T* test and one-way ANOVA). For non-normally distributed data, non-parametric tests (Wilcox- and Kruskal–Wallis tests) were used. Principal Component Analysis (PCA) was used to highlight relations among the composite variables measured in this study. PCA reduced our large data set to a few independent, uncorrelated variables (principal components) that explained much of the variance of the original data. In order to minimize the variation among the variables under each factor, the factor axes were subsequently varimax rotated. As recommended by Tabachnick and Fidell (1996), we interpreted variables with loading >0.45. The software package SPSS 17.0 for Windows was used to complete PCA. The abbreviations of parameters used in this study are listed in Table 2.

**Table 2:** Table of abbreviations used throughout the text

Parameter	Abbreviation
Bacterial abundance	BA
Bacterial secondary production	BSP
Carbon specific UV absorbance	SUVA_254_
CDOM absorption coefficient	a_350_
Chlorophyll *a*	chl *a*
Chromophoric dissolved organic matter	CDOM
Dissolved inorganic nitrogen	DIN
Dissolved organic carbon	DOC
Dissolved organic matter	DOM
Dissolved organic nitrogen	DON
Extent of connection	EC
Extracellular enzymatic index 1	EEA1
Extracellular enzymatic index 2	EEA2
Fluorescence index	FI
Leucin-aminopeptidase	EEAleu
Metabolic effort	MEF
Metabolic effort of leucin-aminopeptidase	MEFleu
Metabolic effort of phenol oxidase	MEFphOx
Metabolic effort of α-glucosidase	MEFa
Metabolic effort of β-glucosidase	MEFb
Phenol oxidase	PhOx
Soluble reactive phosphorus	SRP
Temperature	Temp
α-Glucosidase	EEAa
β-Glucosidase	EEAb

## Results

### Hydrology

**Fig. 2:**
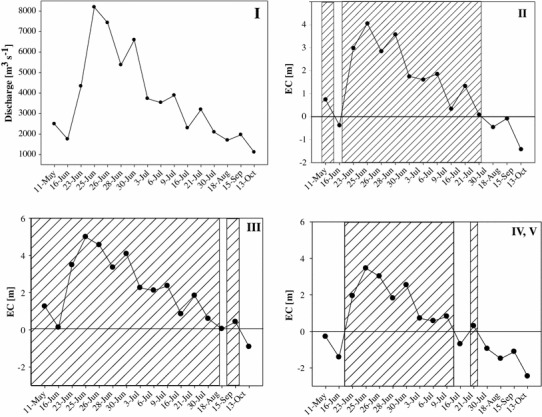
Danube discharge (main channel, I) and extent of connection (EC) for each location (II, III, IV and V) over the sampling period. Two distinct hydrological phases are defined: connected (*lined area*) and disconnected (*white area*)

The year 2009 was characterized by relatively high discharge of the Danube (average 2,222 m^3^ s^−1^), with a 30-year flood event in June. From May until October, 59 sampling events were conducted on 16 dates. Particular focus was placed on the hydrologically most dynamic period (May–August). The repeatedly fluctuating Danube level altered the hydrological conditions in the floodplain backwaters and created a diverse range of surface connections with the main river. Starting in May and continuing until mid-August, the water level was typically above mean water (1,930 m^3^ s^−1^), with discharge values varying from 1,743 to 8,190 m^3^ s^−1^ (Fig. 2I). On 23 June, the first notably higher water level (discharge 4,332 m^3^ s^−1^) occurred, and reached even the most distant locations at the Lobau (Fig. 2II, III, IV). The highest Danube discharge occurred on 25 June. It dropped 5 days after the peak, gradually disconnecting the more remote stations (IV and V) (Fig. 2IV, V). Two additional but smaller rises were noted in July and in August, 4,409 and 4,102 m^3^ s^−1^, respectively. Both also flooded more distant locations (IV and V). Finally, in mid-August, even the frequently flooded section (II) in Lobau became disconnected (Fig. 2II, III).

### Temporal and spatial patterns of DOM quantity and quality

**Fig. 3:**
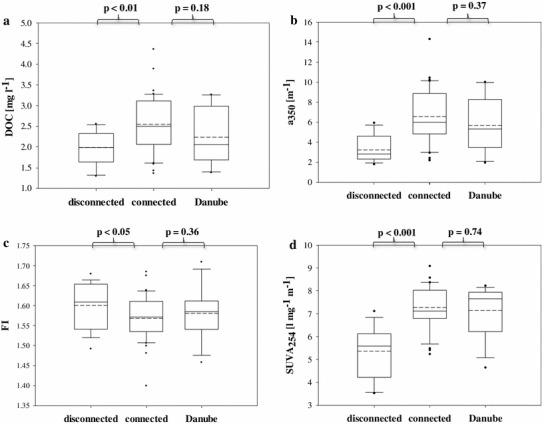
Boxplots of DOM quantity (**a**) and quality (**b**, **c**, **d**) for connected, disconnected conditions and main channel of the Danube. The boundaries of the box plot indicate the 25th and 75th percentiles, points indicate outliers, the *solid line* in the box marks the median, *dashed line* marks the mean. Statistical differences are indicated on the top of each panel. The* t* test was used to check for significant differences in the measured variables between the main channel of the Danube and connected conditions and between connected and disconnected conditions

**Fig. 4:**
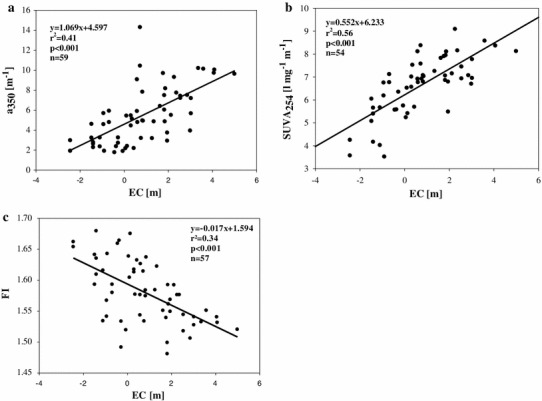
Relationships between hydrology (EC), CDOM quantity (**a**) and DOM quality: SUVA_254_ (**b**) and FI (**c**). For abbreviations see Table 2

During the study, DOM quantity and quality in backwaters was clearly affected by the introduction of the Danube water. During connected conditions, DOC in backwaters increased from 1.36 to 4.37 mg l^−1^ and did not differ significantly from the Danube DOC, which remained in the same range (1.38–3.27 mg l^−1^) (Fig. 3a). During disconnected conditions, however, DOC was significantly lower compared with connected conditions (*p* < 0.01, *n* = 58) (Fig. 3a). The DOC/DON ratio in backwaters increased with the flood, from average 20:1 before the flood, up to 32:1 noted few days after the peak. However, the DOC/DON ratio in the main channel (I) remained lower and stayed in the range of 15:1–21:1. This resulted in significantly lower values of DOC/DON in the Danube compared to backwaters during connected and disconnected phases (*p* < 0.05, *n* = 66). The values of the quantity and quality proxies (a_350_, FI, SUVA_254_) of the backwaters did not differ significantly from the Danube during the connected phases (Fig. 3b–d). However, lack of surface connection with the main channel resulted in significant differences between connected and disconnected phases in the a_350_, SUVA_254_, and FI values (Fig. 3b–d). The CDOM quantity and DOM quality were related to EC, either positively (Fig. 4a, b) or negatively (Fig. 4c). When disconnected conditions were established, DOC concentration was not mirrored by its optical properties, whereas during surface connection with the main channel and in the main channel itself, DOC was significantly negatively correlated with the FI ratio, but positively with SUVA_254_ (Table 3).

**Table 3:** Correlation coefficients between DOM quantity (DOC) and qualitative parameters and selected enzymatic activities; connected (CON), disconnected phases (DISC) and main channel of the Danube

Parameter	DOC		
	DISC	CON	Danube
FI	ns	−0.57**	−0.71**
SUVA_254_	ns	0.65**	0.69*
EEAa	ns	0.46**	0.80**
EEAb	ns	0.46**	0.86**

For abbreviations see Table 2

* *p* < 0.05, ** *p* < 0.01

### Temporal and spatial bacterial parameters

#### Bacterial abundance

**Fig. 5:**
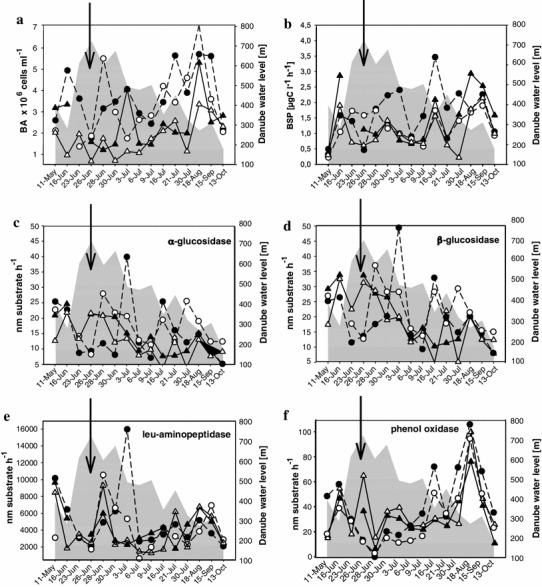
Temporal patterns of bacterial abundance (**a**) and activity parameters: BSP (**b**), α-glucosidase (**c**), β-glucosidase (**d**), leu-aminopeptidase (**e**) and phenol oxidase (**f**) in stations II (*filled triangle*), III (*open triangle*), IV (*filled circle*), and V (*open circle*). *Arrows* indicate peak of the flood in the main channel of the Danube. *Grey area* indicates water level in the main channel of the Danube. For analytical variations and replicates, see “Methods” (analytical variance <5 %), except BSP (10 %)

BA varied over the course of the study with changes in river flow. At the onset of the flood, bacterial numbers decreased, followed by continuous changes until October in a range of 0.41–7.00 × 10^6^ cells ml^−1^ (Fig. 5a). This resulted in significantly higher numbers of bacteria in backwaters during disconnected phases in comparison to the Danube (*p* < 0.05, *n* = 32) and connected phases (*p* < 0.001, *n* = 58).

#### Bacterial production

Bacterial secondary production (BSP) fluctuated considerably within a range of 0.22–3.46 μg C l^−1^ h^−1^ (Fig. 5b). Due to strong fluctuations, there were no significant differences in BSP between connected and disconnected phases, however BSP was generally significantly higher in backwaters during disconnected phases than in the Danube, (*p* < 0.01, *n* = 32).

### Enzyme activities

In general, an initial drop of EEA at flood onset was followed by increased hydrolytic enzyme activity 3–5 days after establishment of the surface connection at stations II, III, IV and V (Fig. 5c–f). Later in the season, hydrolytic enzyme patterns were less consistent at all the locations, with more pronounced fluctuations at rarely connected water bodies. PhOx activity was considerably higher when water bodies were decoupled (*p* < 0.001, *n* = 46), whereas there were no significant differences in hydrolytic enzyme activities between the two different hydrological phases and the Danube main channel (EEAa, *p* = 0.58; EEAb, *p* = 0.24; EEAleu, *p* = 0.88, *n* = 71). Hydrolytic enzyme activity did not change significantly with extent of connection, while PhOx was positively related to EC (*r* ^*2*^ = 0.2, *p* < 0.001, *n* = 57). Activities of EEAa and EEAb were strongly coupled to DOC of the Danube (r = 0.80, r = 0.86, *p* < 0.01) and during connection (r = 0.46, r = 0.46, *p* < 0.01), (Table 3). No correlations were detected between oxidase and hydrolases throughout the season.

A metabolic effort (MEF) parameter [nm of enzyme produced × μg C incorporated^−1^] was calculated by dividing the activity of each enzyme by BSP in order to normalize bacterial enzymatic activity. Since extracellular hydrolysis of the substrate and uptake of the hydrolysis product are strongly related (Chróst and Siuda 2002), the MEF parameter was applied to evaluate the magnitude of enzyme production, which is inversely related to the bacterial carbon assimilation. MEF of all the enzymes fluctuated considerably throughout the season. There were no significant relations between hydrology (EC) and MEF: MEF α-glucosidase (MEFa): *p* = 0.46; MEF β-glucosidase (MEFb): *p* = 0.25; MEF leu-aminopeptidase (MEFleu): *p* = 0.76; MEF phenol oxidase (MEFphOx): *p* = 0.24; n = 59, hence also no significant differences between connected and disconnected situations (MEFa: *p* = 0. 66; MEFb; *p* = 0.88; MEFleu: *p* = 0.78; *n* = 45). The only exception was the significantly higher MEFphOx during disconnection (*p* < 0.05).

Two enzymatic indices were calculated. Although an enzymatic index approach has already been applied in other studies (Clinton et al. 2010; Sinsabaugh et al. 2010), no hydrolytic to oxidative activity has been assessed so far, especially for floodplain backwaters. The ratios of EEAleu to PhOx and EEAa + EEAb to PhOx (further termed extracellular enzymatic index: EEA 1 and EEA 2, respectively) were calculated (Sinsabaugh and Moorhead 1994). They served as proxies to indicate the relative importance of protein and carbohydrate utilization versus lignin degradation for microbial metabolism. EEA 1 and EEA 2 increased with flooding, reaching a maximum at the beginning of the flood. Afterwards, the decrease of indices resulted in significantly lower EEA 1 and EEA 2 (*p* < 0.05, *p* < 0.01, *n* = 57) during the disconnected phase. EEA 1 and EEA 2 were negatively correlated with FI (*r* = −0.35, *r* = −0.39, *p* < 0.05, *n* = 39), but only during connected phases.

**Fig. 6:**
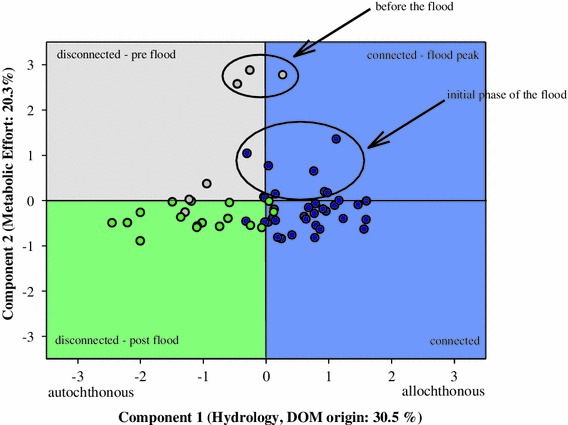
Score plot for the first two principal components from PCA of variables listed in Table 4. The plot was generated from PCA after varimax rotation. Data from disconnected and connected conditions of stations II, III, IV and V were used as PCA input. *Different colours* of the figure quarters indicate distinct hydrological phases. Data points are separated according to the type of connectivity with the main channel. *Grey circles* indicate stations sampled before the flood,* blue circles* during surface connection, *green circles* after disconnection from the main channel. Stations are represented by all parameters listed in Table 4. For more details on PCA see methods and Table 4

A PCA was performed to provide an overview of the patterns and relations within measured parameters during hydrological connection/disconnection phases. For the PCA input, we used the variables listed in Table 4. The variables were calculated for stations II, III, IV and V throughout the sampling season (May–October). Five components were distinguished that explained 81 % of variability in the data. Component 1 accounted for 23.9 % of variability and represented hydrology (EC) and origin of dissolved organic matter (autochthonous vs. allochthonous). Component 1 was positively correlated with EC, a_350_ and SUVA_254_ and negatively with FI. Component 2 explained 23.6 % (Table 4) and illustrated bacterial activity (described as MEF). It was positively correlated with enzymatic investment by heterotrophic bacteria. In Fig. 6 we present an overview of the observed patterns based on PCA by relating it to four distinct hydrological situations. The patterns during surface connection/disconnection phases are illustrated in Fig. 6 and are mainly grouped by component 1 (hydrology and DOM origin); component 2 appears to be less important regarding connection of backwaters to the main channel. The other components were less important, but worth mentioning is component 4, which explained only 12.2 % of variability. Component 4 determined phytoplankton biomass (chlorophyll *a* [chl a]) and inorganic nutrients (Table 4).

**Table 4:** PCA: Explained variation in percent and correlation coefficients of environmental variables with five components

Variable	Correlation coefficients				
	Component 1 (23.86 %)	Component 2 (23.66 %)	Component 3 (12.35 %)	Component 4 (12.28 %)	Component 5 (9.31 %)
EC	**0.851**	0.014	−0.309	0.014	−0.216
FI	−**0.689**	−0.253	−0.028	−0.255	−0.319
SUVA_254_	**0.916**	0.102	0.077	−0.188	−0.067
a_350_	**0.861**	−0.109	−0.024	−0.222	0.199
DOC/DON	0.020	0.076	0.023	−0.109	**0.931**
DIN/SRP	−0.048	0.119	0.145	**0.878**	−0.014
Temp	0.126	−0.009	**0.919**	−0.051	0.012
Chl *a*	−0.363	−0.046	−0.013	**0.573**	−0.327
MEFa	0.110	**0.937**	−0.063	0.005	0.136
MEFb	0.137	**0.949**	−0.095	0.005	0.112
MEFleu	0.013	**0.850**	−0.055	0.183	−0.053
MEFphOx	−0.191	**0.670**	0.169	−0.502	−0.097
BA	−0.323	−0.128	**0.769**	0.218	0.017

Boldface: correlation coefficients >0.5. Bartlett’s Test of Sphericity was *** *p* < 0.001. For abbreviations see Table 2. For more details see “Methods”

## Discussion

### Importance of surface connectivity for DOM heterogeneity

Our results confirm that the flood introduced into the backwaters DOM that consisted of more allochthonously derived, aromatic compounds, as has also been found in other rivers (Guéguen et al. 2006) and wetlands (Hunsinger et al. 2010). During the floods, inputs of allochthonous DOC in streams are under hydrological control (Royer and David 2005), and elevated discharge is often related to higher DOC concentrations (Guéguen et al. 2006; Mladenov et al. 2007; Peduzzi et al. 2008).

In our study, the flood also introduced significantly more DOC, but more interestingly, the optical properties, related to EC (Fig. 4a–c) suggest that hydrological events altered DOM composition in backwaters substantially. Positive relations between DOC and SUVA_254_, and negative with FI (Table 3) in the Danube and during connected phases, indicate that DOC in the floodplain backwaters was mostly derived from the main channel and contained a high proportion of chromophoric, allochthonous and aromatic material. This agrees with previous reports indicating hydrological control of the FI (Peduzzi et al. 2008; Besemer et al. 2009). However, the lower DOC/DON values in I, compared to DOC/DON during connected phases in backwaters, support the idea that the floodplain itself is a potential DOM source during flood conditions (Mladenov et al. 2005). The above findings suggest that the DOM was a mixture of river and floodplain-derived DOM. Nonetheless, we could not observe that the flood was a source of very bioavailable DOM, since its chemical properties pointed to a refractory nature of introduced material.

At disconnection, we recorded significantly lower SUVA_254_, which denotes that the DOM pool during the disconnected phase is composed of less aromatic molecules. Significantly higher FI implies the introduction of other, most likely autochthonous material to the DOM pool of the floodplain system (Peduzzi et al. 2008; Besemer et al. 2009). Note that the increase in FI could also reflect the removal of allochthonous organic matter in conjunction with microbial/autochthonous production.

### EEA as a tool to track alterations in DOM availability

Responses to changes in DOM sources can be traced by examining bacterial enzymatic activity (Clinton et al. 2010), by linking EEA to DOC composition (Burns and Ryder 2008). Within a bacterial community, the composition of extracellular enzymes may shift in relation to changing sources of DOM. Accordingly, enzyme activity patterns imply potential availability of different DOM components (Chróst 1991). EEAb activity is repressed by the presence of readily utilizable bacterial substrates (i.e. glucose), whereas the appearance of more complex saccharides strongly induces EEAb activity (Chróst 1991). Pronounced temporal peaks and significant correlation of DOC with EEAa and EEAb (Table 3) point to rapid utilization of available carbon sources (Burns and Ryder 2008). Simultaneous increase of EEAa and EEAb right after the flood peak (Fig. 5c, d) indicates introduction of more complex, storage and structural polysaccharides (Sinsabaugh and Foreman 2001). The quick response of glucosidases implies that even short pulses stimulate activity of bacterial glucosidases (Burns and Ryder 2008). The delayed, but pronounced, EEAleu reaction (5–11 days) (Fig. 5e) shows that longer floods promote hydrolysis of proteins and demonstrates that the introduction also of bioavailable DOM was linked to the flood (Stepanauskas et al. 2000). We suggest that, investigation of EEA during rapid environmental changes is a very useful tool to assess the reactivity of DOM. Although results of the optical properties pointed to a more aromatic and refractory nature of DOM during connected phases, the EEA approach revealed that some introduced material was bioavailable and could be utilized by the bacterial community. The pronounced changes in EEA point to the presence of bioavailable polysaccharides introduced with the flood event. Some of the polysaccharides are not chromophoric and they would not be detected based on optical properties. Therefore, previous studies, which relied solely on DOM optical properties, might have missed the mobilization of non-chromophoric, but bioavailable and ecologically important polysaccharides and proteins.

Despite extensive studies on hydrolytic enzyme activity, less is known about PhOx in aquatic environments. This enzyme degrades recalcitrant, aromatic, phenolic compounds such as lignin or humic material into more readily available substrates (Sinsabaugh 2010). Fungi are responsible for initial degradation of native lignin, while bacteria are considered to play a main role in mineralization of lignin-derived low-molecular weight compounds in soils (Masai et al. 2007). However, in aquatic ecosystems, bacteria seem to play a leading role in decomposing lignin (Li et al. 2008). The significant increase of phenol oxidase associated with lignin degradation (DeAngelis et al. 2011) typically is a longer process compared to the quicker response of glucosidases (Smart and Jackson 2009). PhOx and glucosidases are usually decoupled, (as shown in our study) which suggests different controlling mechanisms on these two classes of enzymes (Sinsabaugh 2010).

Most PhOx studies focused on activity in soil and litter (Pind et al. 1994; Williams et al. 2000; Sinsabaugh 2010), whereas activity in inland waters is less explored (Münster and De Haan 1998). Factors that control PhOx activity in soil include pH, concentration of soluble phenolic compounds, lignin content of plant litter, along with oxygen and nitrogen availability (Sinsabaugh 2010). Our results showed that PhOx was influenced by hydrology, with significantly higher activity during disconnected phases. This suggests that hydrological changes such as flooding and subsequent lowering of the water table promoted activity of PhOx. Flooding can strongly stimulate loss rates of lignin in soils (Lockaby et al. 1996), whereas in ponds, activity of PhOx may be triggered after flooding (Alvarez and Guerrero 2000). Nonetheless, there is some discrepancy about the response of PhOx to water level changes. Freeman et al. (2001) reported a significant increase as the water table dropped in peatlands, whereas Williams et al. (2000) found no relation of PhOx to wetland hydrology. Additionally, our results show that during the disconnection, activity of phOx was promoted by aromatic content of DOM (*r* = 0.48, *p* < 0.05), which points to bacterial utilization of lignin-derived aromatic compounds (Masai et al. 2007) during disconnected phases. Significantly higher PhOx after the flood also implies that degradation of aromatic material could be responsible for significant decrease of SUVA_254_ after the flood.

### Effect of surface connectivity on the fate of utilized DOM

Our results confirm that DOM quality is an important factor regulating bacterial production in floodplain areas (Peduzzi et al. 2008). Typically, DOM of autochthonous origin is higher-quality material that stimulates BSP (Cole et al. 2002), as opposed to allochthonously derived DOM, which is recalcitrant with much longer turnover times (del Giorgio and Pace 2008). At the same time, there is evidence that BSP can also be controlled by allochthonous organic carbon rather than by within-system primary production alone (Tranvik 1992; Kritzberg et al. 2004; Daniel et al. 2005). One suggestion is that allochthonous material supports almost exclusively respiration, whereas algae prevailingly support carbon assimilation (Cole et al. 2002; Kritzberg et al. 2005; Karlsson et al. 2007). Nonetheless, abundant allochthonous DOM input may not only boost respiration but also increase bacterial production (Thottathil et al. 2008).

In order to describe DOM utilization patterns and quality during connected and disconnected phases, we applied new parameters: MEF, EEA 1 and EEA 2, (see “Results”). MEF serves as an assimilation efficiency parameter that indicates the proportional importance of catabolic versus anabolic processes. Lower values indicate that substrate, which undergoes enzymatic degradation, drives bacterial metabolism towards anabolism. We did not measure bacterial respiration, however with PCA analysis (Fig. 6), where we incorporated the MEF parameter, we attempted to explain the importance of catabolic versus anabolic processes. Our results of the PCA demonstrate (Table 4) that hydrological disturbances and DOM quality (Component 1) appear to be crucial in the functioning of this system. The MEF parameter loaded higher on Component 2 (Fig. 6; Table 4), and MEFa and MEFb were significantly higher in dynamic stations (II, III) (*p* < 0.05, n = 19) during the first 7 days of the flood. These results imply that the onset of the flood boosts catabolic processes (del Giorgio and Pace 2008). At the same time, the lack of significant differences of MEFa, MEFb and MEFleu between connected and disconnected phases indicates that prolonged connectivity with the main channel only minimally influences the assimilation efficiency of hydrolyzed products (Fig. 6). What is surprising is the pre-flood situation: despite the few measurements, the degraded substrates (even of autochthonous origin) generate less bacterial biomass per energy expenditure (Fig. 6). Significantly higher MEFphOx during disconnected conditions supports the conclusion that longer water retention promotes this enzymatic activity per unit of production (Sinsabaugh 2010). This calls for studying the factors that determine elevated MEFphOx in floodplain waters during disconnected phases.

Although we did not conduct photodegradation experiments, photodegradation may be important during disconnected phases. The flood introduced significantly higher amounts of aromatic and chromophoric material. Such material is considered to be photo-labile DOM (Stubbins et al. 2010) and may be substantially photo-altered in the post-flood period (less turbid conditions). However, the issue of photodegradation in our system remains to be investigated.

### Bacterial utilization of high vs. low reactive DOM

The EEA 1 and EEA 2 indicate which part of the DOM pool (high/low reactivity DOM) was a dominant component for bacterial metabolism. This enables assessing bioavailability and quality of material from different sources. The substantial increase of the EEA 1 and EEA 2 in all the backwaters, with a maximum at the 5th day of connection, indicates a switch to polysaccharide and protein metabolism. The negative relation of EEA 2 with the FI during connected phases suggests that microbial production of glucosidases is induced by allochthonous DOM. Therefore, we suggest that DOM introduced with this flood water is more bioavailable than previously thought and contains reactive organic matter that is processed relatively quickly. High aquatic BSP during and shortly after floods may be supported by the more bioavailable fraction of the small, readily available sub-pool of the terrestrial DOM that is flushed from the surrounding area (Ågren et al. 2008).

**Fig. 7:**
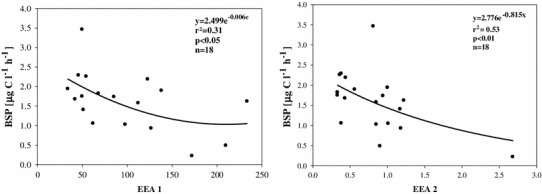
BSP as a function of extracellular enzymatic indices (EEA 1 and EEA2) during disconnected phases. For abbreviations see Table 2

In floodplains, during seasonal floods, rapidly decaying plant-derived sources (terrestrial and aquatic) dominate the DOM pool. In contrast, non-flood periods are better described by the presence of a more slowly processed pool (Mladenov et al. 2007). In our study, a switch from protein and polysaccharide (highly reactive) to lignin (low reactive) degradation was indicated by significantly lower EEA 1 and EEA 2 during the disconnected phase. An increased importance of lignin material for heterotrophic metabolism is further supported by significant negative relations between BSP and EEA 1 or EEA 2 (Fig. 7) during disconnection. Microbial degradation of lignin material occurs constantly throughout the water column and is an important process in removing dissolved lignin phenols (Hernes and Benner 2003). Although this semi-labile pool is used up more slowly (days-months) (Benner and Kaiser 2011), it still supports some level of bacterial metabolism (del Giorgio and Pace 2008).

Although carbon flux in water-linked landscapes is still poorly understood, floodplains play a significant role in carbon sequestration and mineralization processes (Battin et al. 2008). Due to the reduced hydrologic connectivity in human-impacted, regulated rivers, numerous services and functions of floodplains are impaired (Tockner et al. 2006). This includes the reduced opportunity to process large amounts of organic carbon, severely alterating the overall carbon flux. Our results stress the importance of seasonal hydrological events that connect a river channel with adjacent floodplains (Schiemer et al. 2006). Flooding mobilized a significant fraction of the reactive DOM from the catchment, which then subsidizes bacterial metabolism (Carpenter et al. 2005); sudden and increased amounts of different carbon sources can promote microbial productivity (Docherty et al. 2006). Thus, terrestrial material may be also processed quickly (Lam et al. 2007), and carbon flow through bacteria can in certain situations be dominated by allochthonously derived DOM (Carpenter et al. 2005). Therefore, allochthonous DOM imported during floods is important for BSP (Berggren et al. 2009). Interestingly, utilization of different C sources does not necessarily substantially affect the proportions of energy expenditure versus microbial biomass generation. The minor influence of prolonged connectivity on the assimilation efficiency of hydrolyzed products could be explained by a very adaptive metabolic capacity of the bacterial community (compare Fig. 6). Within the frame of physiological possibilities, the bacterial assemblage may respond with enzymatic and/or community composition changes.

Thus metabolic changes can be based on the metabolic flexibility of ecotypes of specific species, or be a result of changes in community structure. Nonetheless, the changed conditions for processing of allochthonous DOM, when residing in backwaters, provide different opportunities for carbon processing in floodplains. The existence of such a river-floodplain connection should also be important for downstream carbon transport. Further studies are necessary to better understand the share of the DOM pool in bacterial C assimilation versus respiration during/after flood events.
